# Characterization and Detection Strategy Exploration in Cryptogenic Hepatocellular Carcinoma: Insights From a Super‐Aged Region in Japan

**DOI:** 10.1002/cam4.70490

**Published:** 2025-01-23

**Authors:** Takaaki Sugihara, Takakazu Nagahara, Takuya Kihara, Suguru Ikeda, Yoshiki Hoshino, Yukako Matsuki, Takuki Sakaguchi, Hiroki Kurumi, Takumi Onoyama, Tomoaki Takata, Tomomitsu Matono, Hajime Isomoto

**Affiliations:** ^1^ Division of Gastroenterology and Nephrology, Department of Multidisciplinary Internal Medicine, Faculty of Medicine Tottori University Yonago Japan; ^2^ Department of Gastroenterology Hyogo Prefectural Harima‐Himeji General Medical Center Himeji Japan

**Keywords:** cryptogenic, epidemiology, hepatocellular carcinoma, surveillance

## Abstract

**Background and Aim:**

In recent years, there has been a rise in cryptogenic hepatocellular carcinoma (c‐HCC) cases in Japan, posing a detection challenge due to an unknown etiology. This study aims to enhance diagnostic strategies for c‐HCC by analyzing its characteristics and exploring current opportunities for detection.

**Methods:**

A retrospective study was conducted from April 2012 to March 2022, enrolling 372 newly diagnosed hepatocellular carcinoma (HCC) patients. Excluding cases associated with hepatitis viral infection, alcoholic liver disease, non‐alcoholic fatty liver disease/steatohepatitis, autoimmune hepatitis, primary biliary cholangitis, and congestive hepatopathy, the study specifically focused on genuine c‐HCC. The analysis delved into the characteristics, detection opportunities, and survival outcomes associated with c‐HCC.

**Results:**

Among the non‐viral HCC cases, 55 patients (29.3%) (34 men and 21 women) were diagnosed with c‐HCC, making it the second‐highest etiology. Notably, individuals with c‐HCC, typically aged 60 and above (median age 76.0), exhibited a women predominance and presented with larger tumors (4.5 cm vs. 2.5 cm), correlating with a poorer prognosis. Cirrhosis was notably absent in most c‐HCC cases (72.7%), and more than half (56.4%) did not have diabetes mellitus (DM). Diagnostic pathways for c‐HCC primarily involved incidental imaging (47%) and symptoms (24%). Within the cohort of c‐HCC, the prognosis for symptomatic cases is notably unfavorable compared to other cases [median survival time 19.0 (7.0–45.0) months vs. 47.0 (29.0–76.0) months, *p* = 0.029]. In the multivariate regression analysis, age and women emerged as independent factors associated with c‐HCC. Rather than a significant increase in women, there is a narrowing gender gap.

**Conclusion:**

Patients with c‐HCC were predominantly elderly, without cirrhosis or diabetes, and exhibited minimal gender differences. Detection often occurred incidentally through abdominal imaging. Considering the limitations of conventional surveillance, it seems reasonable to propose that abdominal imaging be included in cancer screening, particularly for individuals aged 60 and older.

**Trial Registration:**

1610A127

## 1 Introduction

Primary liver cancer is the third leading cause of cancer death worldwide in 2020, with approximately 830,000 deaths, according to GLOBOCAN 2020 data [1]. Hepatocellular carcinoma (HCC) comprises 75%–85% of primary liver cancers. The main risk factors for HCC development are infection by hepatitis B virus (HBV) and hepatitis C virus (HCV). Tremendous advances have been made over the decades, and HCV became the first curable, chronic viral hepatitis [2]. Chronic HBV infection remains a major global health burden. The currently available treatments for HBV infection‐interferon and nucleos(t)ide effectively suppress viral replication and decrease the risk of cirrhosis [3]. However, the incidence of HCC, which is negative for both markers of HBV and HCV infection [non‐B non‐C (NBNC)], has recently increased in Japan [4]. Such non‐viral HCC comprises non‐alcoholic fatty liver disease/ steatohepatitis (NAFLD/NASH), alcoholic liver disease (ALD), and others. Researchers have tried establishing an enclosure strategy for NAFLD/NASH‐HCC for decades.

On June 24, 2023, the European Association for the Study of Liver (EASL), the American Association for the Study of Liver Diseases (AASLD), and the Latin American Association for the Study of the Liver (ALEH) jointly introduced the concept of metabolic dysfunction‐associated steatotic liver disease/steatohepatitis (MASLD/MASH), MetALD, and ALD. This change is expected to broaden the coverage of non‐viral HCC cases [5].

Except for MASLD/MASH and known etiologies, the residual category will be classified as cryptogenic‐HCC (c‐HCC). Gaining an in‐depth understanding of c‐HCC, which remains out of the loop in the non‐viral HCC arena, would be crucial to eradicating HCC. However, it should be noted that the definition of c‐HCC differs among reports and changes over time. In a report from the 1990s, the definition of c‐HCC included conditions such as ALD and autoimmune hepatitis (AIH) and did not yet encompass NAFLD [6, 7]. More recent reports commonly define c‐HCC as a condition characterized by the absence of hepatitis B, hepatitis C, or ALD [8, 9, 10]. The incidence of c‐HCC reportedly accounts for approximately 7%–9% of HCC cases [7, 9, 10]. Reports indicated that c‐HCC is associated with older age at diagnosis, more frequent occurrence of metabolic syndrome, and less aggressive tumor characteristics, but similar survival compared to viral or alcoholic HCC [9]. Established etiological factors like NAFLD/NASH, AIH, primary biliary cholangitis (PBC), primary sclerosing cholangitis (PSC), or congestive hepatopathy can be currently encompassed within surveillance. Our focus, however, is on genuine c‐HCC cases that lack identifiable etiologies.

Thus, in this study, we aimed to elucidate the characteristics of genuine c‐HCC, excluding known etiologies, and to identify key indicators for its detection.

## 2 Patients and Methods

### 2.1 Patient Selection Criteria

This was a single‐center, retrospective, observational study involving 417 patients from our hospital diagnosed with HCC that examined alpha‐fetoprotein (AFP) and des‐gamma‐carboxy prothrombin (DCP) at baseline between April 2012 and March 2022. Patients who took warfarin, vitamin K, or antibiotics were excluded because they would influence DCP levels. Among the patients whose histopathology was unavailable, we selected only those diagnosed with typical HCC according to LR ≥ 4 (arterial phase hyperenhancement) on the CT/MRI Liver Imaging Reporting and Data System LI‐RADS v2018 [11]. In this study, Barcelona Clinic Liver Cancer (BCLC) stages and TNM (UICC eighth edition) were used [12, 13]. The Albumin‐Bilirubin (ALBI) score in this study was determined using the following formula: log_10_ bilirubin (μmol/L) × 0.66—albumin (g/L) × 0.085 [14]. A modified ALBI (mALBI) grade, as outlined in previous literature, was employed in this study [15, 16, 17]. Patients without viral hepatitis (HBsAg and HCV‐Ab negative) were classified as non‐viral HCC. Patients with hepatic steatosis diagnosed with imaging (ultrasonography, US; computed tomography, CT; or magnetic resonance imaging, MRI) with any of the cardiometabolic criteria were classified as MASLD/MASH according to the new definition introduced by AASLD [18]. The definition of hepatic steatosis by imaging was described in our previous report [19]. In this study, patients who had alcohol consumption exceeding 30 g per day for men and 20 g per day for women were all classified in the Alcohol group, which includes both MetALD and ALD. The diagnoses of AIH, PBC, and PSC were made based on the respective Japanese guidelines: AIH according to the 2021 revision [20], PBC according to the 2023 revision, which is available only in Japanese and maintains the same diagnostic criteria as the previous version [21], and PSC according to the clinical guidelines [22]. The definition of hepatic congestion requires the presence of any pre‐existing chronic right ventricular heart failure, including a history of undergoing the Fontan procedure [23]. Patients with these known etiologies (alcohol, AIH, PBC, PSC, MASLD/MASH, and congestive hepatopathy) were excluded. The staging of liver fibrosis is primarily confirmed through histological examination, while the remaining cases are evaluated using imaging techniques, such as contrast‐enhanced CT or MRI, in accordance with established diagnostic criteria [24]. Key indicators include identifying morphological changes in the liver, encompassing irregularities in the liver surface, hypertrophy of the caudate lobe, atrophy of the right hepatic lobe, and the presence of portal hypertension, including splenomegaly. In the imaging‐only patients, cirrhosis was diagnosed based on a comprehensive evaluation using vibration‐controlled transient elastography (VCTE) with a cutoff value of 15 kPa and/or a platelet count below 150 × 10^3^/mL, as per the criteria for compensated advanced chronic liver disease (cACLD) outlined in Baveno VII [25]. Those testing positive for HBc‐Ab were excluded as having occult HBV infection (OBI).

### 2.2 Statistical Analysis

Statistical comparisons between two independent groups utilized the Mann–Whitney test or chi‐square test. For cases involving more than three groups, the Kruskal–Wallis test was applied, followed by post hoc analysis using Dunn's test with Bonferroni correction. Multivariate regression analysis was employed to identify independent factors associated with c‐HCC. Statistical analyses were conducted using StatFlex (Windows ver. 7.0; Artech, Osaka, Japan). Cumulative survival rates were calculated using the Kaplan–Meier method. In cases where the observation period was less than 1 month, they were excluded from the survival analysis. The Kaplan–Meier curves were drawn using the Python package “lifelines” package (version 0.27.8). To compare the survival distribution, we performed the log‐lank test utilizing StatFlex (Windows ver. 7.0; Artech, Osaka, Japan). Values are presented as mean ± SD or median (95% confidence interval). Median survival time (MST) with a 95% confidence interval was utilized for survival analysis. Patients with a follow‐up period of less than 1 month and insufficient follow‐up data after the intervention were excluded from the analysis due to the inability to accurately evaluate survival rates and treatment effects. To facilitate a comparison between the results of the analysis including the excluded patients and those excluding them, we have also provided the findings from the analysis that included all patients, referred to as PEA (Presentation of the Entire Analysis). Notably, no patients were excluded in the c‐HCC cohort. Statistical significance was set at *p* < 0.05.

## 3 Results

### 3.1 Patient Selection

**FIGURE 1:**
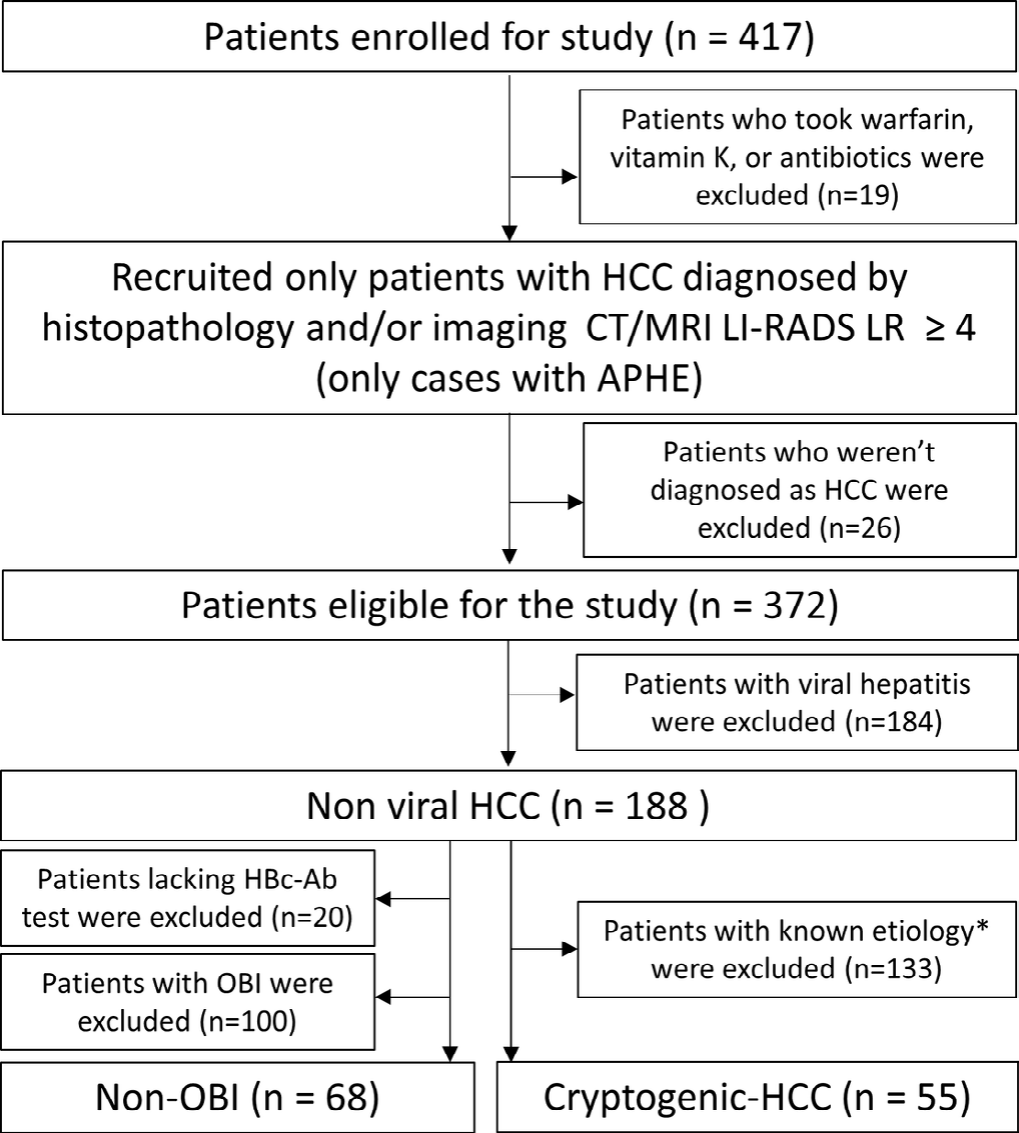
Flowchart of patient selection. At the outset, 417 patients diagnosed with HCC were initially enrolled, yet 19 were subsequently excluded due to their use of warfarin or antibiotics. Only patients with HCC diagnosed by histopathology and/or imaging were selected. Finally, 372 patients were selected for this study. By excluding individuals with viral hepatitis, 188 patients were diagnosed with non‐viral HCC, and 55 patients were diagnosed with cryptogenic HCC after excluding those with known etiologies. Twenty without an HBc‐Ab test were excluded. HBc‐Ab‐positive patients were excluded as OBI. Finally, 68 patients were identified as non‐OBI. LI‐RADS, Liver Imaging Reporting, and Data System; APHE, arterial phase hyperenhancement; OBI, occult hepatitis B virus infection. *Known etiologies include alcoholic liver disease, autoimmune hepatitis, primary biliary cholangitis, metabolic dysfunction‐associated steatotic liver disease/steatohepatitis, and Fontan‐associated liver disease.

Initially, 417 patients diagnosed with HCC were included, but 19 were subsequently excluded due to the use of warfarin or antibiotics. Only patients with HCC confirmed by histopathology and/or imaging were included, with histopathology used for the diagnosis in 172 patients. In the end, 372 patients were selected for the study. After excluding individuals with viral hepatitis, 188 patients were diagnosed with non‐viral HCC. An additional 20 patients were excluded due to the absence of an HBc‐Ab test. 100 patients with positive HBc‐Ab were excluded as OBI. Ultimately, 68 patients were identified as non‐OBI. Finally, 55 patients were identified with c‐HCC after excluding those with known etiologies (Figure 1).

### 3.2 Characteristics of Patients With c‐HCC

**FIGURE 2:**
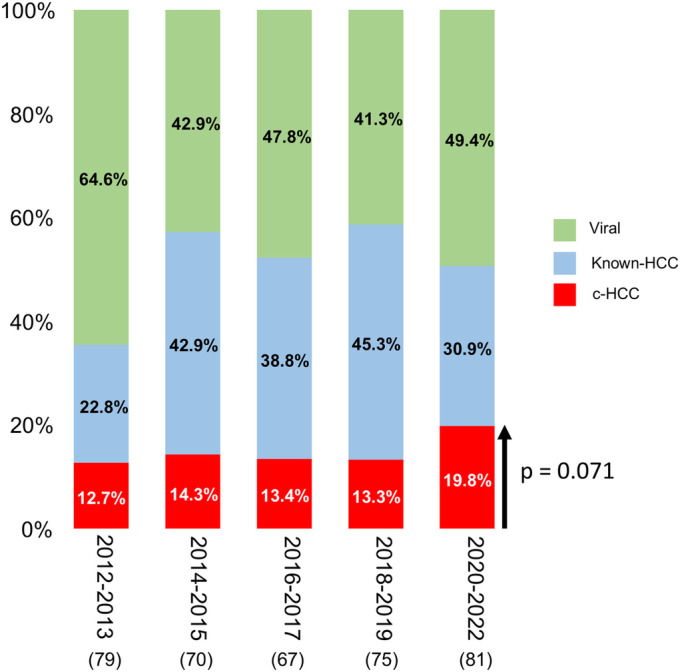
Distribution of disease etiology among new hepatocellular carcinoma cases in each period. HCC, hepatocellular carcinoma; c‐HCC, cryptogenic hepatocellular carcinoma; Known‐HCC, hepatocellular carcinoma of known etiologies. Numbers in parentheses represent the count of patients. Between 2020 and 2022, there has been an upward trend in the proportion of c‐HCC, reaching 19.8% when classified into five periods spanning from 2012 to 2022 (*p* = 0.071).

In recent years, there has been an upward trend in the proportion of c‐HCC, reaching 19.8% when classified into five periods spanning from 2012 to 2022 (*p* = 0.071) (Figure 2).

**FIGURE 3:**
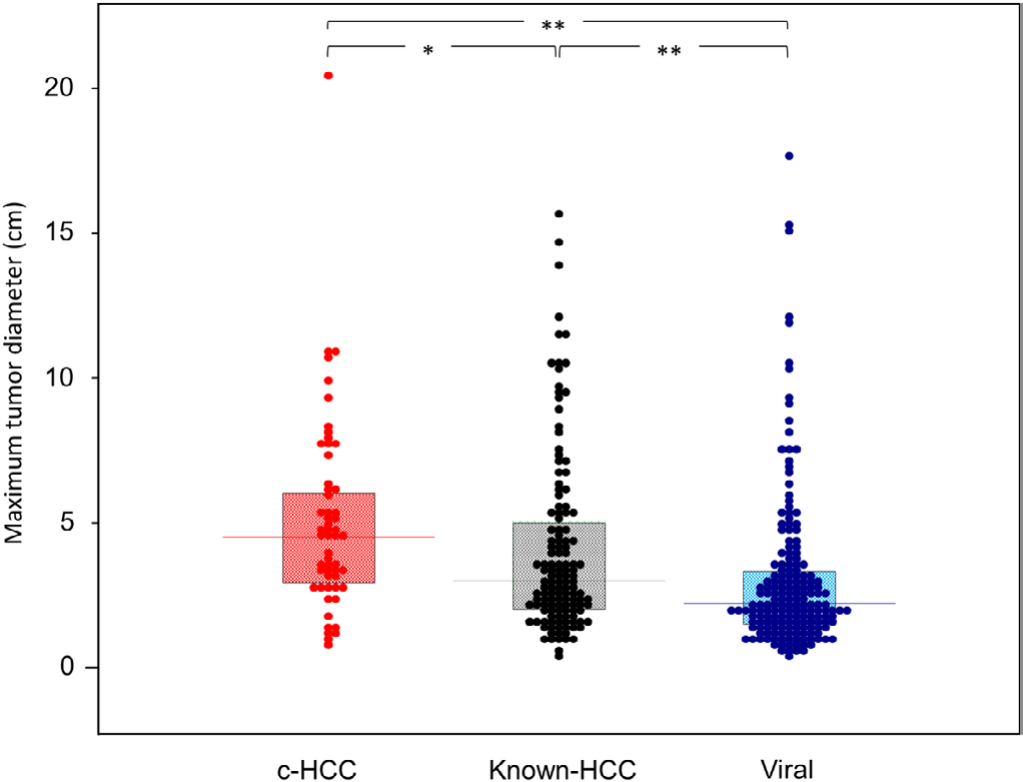
The differences of maximum tumor diameter: A comparative analysis between HCC with Known‐HCC and c‐HCC. In the cases of c‐HCC, the maximum tumor diameter was significantly larger than the others. c‐HCC, cryptogenic hepatocellular carcinoma; Known‐HCC, hepatocellular carcinoma of known etiologies. *p < 0.05 **p < 0.01.

Patient characteristics are shown in Table 1. We enrolled 372 patients [283 men, 89 women, 71.4 ± 10.4 years] in this study. The causes of their liver diseases included viral (*n* = 184, 49.5%) and non‐viral (*n* = 188, 50.5%). Non‐viral HCC includes Alcohol (*n* = 108, 57.4%), MASLD/MASH (*n* = 18, 9.5%), AIH (*n* = 3, 1.5%), PBC (*n* = 3, 1.5%), congestive hepatopathy (*n* = 1, 0.5%), and c‐HCC (*n* = 55, 29.3%). We defined HCC of known etiologies, including Alcohol, MASLD/MASH, AIH, PBC, and congestive hepatopathy, as “Known‐HCC.” One case of congestive hepatopathy was diagnosed as Fontan‐associated liver disease (FALD). The median age of c‐HCC was significantly higher than viral [76.0 (53.3–92.0) vs. 71.0 (46.0–86.0) years., *p* < 0.001] or Known‐HCC [76.0 (53.3–92.0) vs. 72.0 (55.3–85.2) years., *p* = 0.006] and significantly higher in women (37.5% vs. 10.6%, *p* < 0.001). The body mass index (BMI) was significantly higher in Known‐HCC than in cases of viral‐induced HCC [23.1 (17.5–32.8) vs. 24.0 (18.2–32.9) kg/m^2^, *p =* 0.011]. However, in c‐HCC patients, the median BMI did not differ from Known‐HCC [23.9 (17.9–34.7) vs. 24.0 (18.2–32.9) kg/m^2^, *p* = 0.435], and more than half (56.4%) did not have diabetes mellitus (DM). The BMI of Known‐HCC cases was significantly higher than viral‐induced HCC cases. The staging of liver fibrosis was confirmed by histology in 123 patients (33.1%), including 21 with c‐HCC. The remaining patients were diagnosed using contrast‐enhanced CT in 63.4% of cases and MRI in 4%, combined with VCTE and platelet counts. In the patients of c‐HCC, only 15 patients (27.3%) exhibited cirrhosis, indicating a significantly lower incidence than the remaining HCC groups (*p* < 0.001). The lower incidence of cirrhosis would be reflected in the higher platelet concentration in c‐HCC than in the others. There were no statistical differences among the three groups in the Child‐Pugh classification in cirrhosis; however, the mALBI grade was significantly lower in viral‐induced HCC and higher in Known‐HCC. In virus‐induced HCC, there were a greater number of cases in the early stages (TNM IA, BCLC‐0), while in c‐HCC, they were significantly fewer. No patients were detected in BCLC‐0 in c‐HCC. The maximum tumor size was significantly larger [4.5 (1.1–12.1) cm] in c‐HCC than in Known‐HCC [3.0 (1.0–12.4) cm, *p* = 0.028] and viral‐induced HCC [2.2 (0.8–11.9) cm, *p* < 0.001] (Figure 3). Notably, viral‐induced HCC exhibits a significantly better prognosis, attributable to its detection at an earlier stage and feasibility of locoregional therapy. Conversely, the c‐HCC group had significantly lower utilization of locoregional therapy than the others (*p* = 0.002). Therefore, the complete remission (CR) rate was also significantly lower than the others (*p* = 0.003). When locoregional therapy was not indicated, hepatic arterial infusion chemotherapy (HAIC) (*n* = 24), systemic chemotherapy (*n* = 21), and best supportive care (BSC) (*n* = 6) were applied. Lenvatinib was the most frequently employed for systemic chemotherapy (*n* = 15), while immune checkpoint inhibitors were used in only four cases of viral‐induced HCC. Survival was significantly better in c‐HCC treated with chemotherapy (HAIC or systemic) compared to viral‐induced HCC [22 (11.0–45.0) months vs. 5 (4.0–26.0) months, *p* = 0.017]. The mALBI grade was also significantly better in c‐HCC (*p* = 0.010).

**TABLE 1:** Patient characteristics.

Characteristics	Total (*n* = 372)	Viral (*n* = 184)	Non‐viral HCC (*n* = 188)		*p*
			c‐HCC (*n* = 55)	Known‐HCC (*n* = 133)	
Men/Women	283: 89	130: 54	34: 21	119: 14	< 0.001
Age (years)	72 (65.0–88.0)	71.0 (46.0–86.0)	76.0 (52.3–92.0)	72.0 (55.3–85.2)	< 0.001
BMI	23.5 (17.6–33.1)	23.1 (17.5–32.8)	23.9 (17.9–34.7)	24.0 (18.2–32.9)	0.012
DM			24 (43.6%)	66 (49.6%)	0.455
Etiology					
HBV infection	83	83			
HCV infection	99	99			
HBV+HCV	2	2			
Alcohol^a^	108			108	
MASLD/MASH	18			18	
AIH	3			3	
PBC	3			3	
Congestive hepatopathy	1			1	
Blood tests					
Platelet (x10^3^/mL)	151.2 ± 69.1	144.9 ± 65.6	179.0 ± 70.9	148.2 ± 70.9	0.006
AST (U/L)	50.6 ± 49.0	53.2 ± 55.4	42.3 ± 35.6	50.3 ± 44.1	0.426
ALT (U/L)	41.6 ± 40.4	42.4 ± 36.8	36.8 ± 39.3	42.3 ± 45.3	0.098
Alb (g/dL)	3.79 ± 0.59	3.88 ± 0.58	3.78 ± 0.58	3.67 ± 0.58	0.001
FIB‐4 index	4.63 ± 4.03	4.81 ± 4.69	3.68 ± 2.44	4.77 ± 3.51	0.132
Liver status					
Cirrhosis	173	83 (45.1%)	15 (27.3%)	77 (57.9%)	< 0.001
CP‐A	115	59	10	48	
CP‐B	51	21	5	25	0.661
CP‐C	7	3	0	4	
mALBI grade					
Grade 1	182	103	29	50	
Grade 2a	73	36	13	24	0.002
Grade 2b	96	34	10	52	
Grade 3	21	11	3	7	
Tumor markers					
AFP	6.6 (1.3–16884.8)	6.3 (1.2–18569.9)	9.0 (2.1–21898.5)	6.0 (1.4–25827.6)	0.108
DCP	75.0 (11.8–65674.8)	44.0 (13.0–32074.8)	198.0 (16.4–300607.4)	88.0 (10.0–141894.5)	< 0.001
Imaging diagnosis					
Typical HCC^b^	364	180 (97.8%)	54 (98.2%)	130 (97.7%)	0.982
Histologically proven HCC	172 (46.2%)	74 (40.2%)	33 (60.0%)	65 (49.0%)	
Well‐differentiated	58	27	9	22	
Moderately differentiated	103	40	20	43	0.079
Poorly differentiated	11	7	4	0	
BCLC staging					
0	92	66	5	21	
A	164	76	23	65	
B	52	17	13	22	< 0.001
C	54	22	13	19	
D	10	3	1	6	
TNM staging					
IA	91	64	5	22	
IB	123	60	21	42	
II	91	35	11	45	
IIIA	32	8	10	14	< 0.001
III B	14	8	3	3	
IVA	2	2	0	0	
IVB	19	7	5	7	
*First treatment* ^c^					
Locoregional therapy					
RFA	137	86	8	43	
Resection	110	53	20	37	
TACE	58	22	12	24	
SBRT	3	2	0	1	
Others					
HAIC	24	10	6	8	0.002^d^
Systemic^e^	21	4	7	10	
BSC	16	5	2	9	
CR to the first treatment	284 (76.3%)	153 (84.1%)	36 (65.5%)	95 (71.4%)	0.003

*Note:* Data expressed as median (95% CI) or mean ± SD.

Abbreviations: AFP, alpha‐fetoprotein; BCLC, Barcelona Clinic Liver Cancer; BMI, body mass index; BSC, best supportive care; CP, Child‐Pugh classification; CR, complete response; DCP, des‐gamma‐carboxyprothrombin; DM, diabetes mellitus; HAIC, hepatic artery infusion chemotherapy; HBV, hepatitis B virus; HCC, hepatocellular carcinoma; HCV, hepatitis C virus; mALBI, modified ALBI; MASLD/MASH, metabolic dysfunction‐associated steatotic liver disease/steatohepatitis; RFA, radiofrequency ablation; SBRT, stereotactic body radiotherapy; TACE, trans‐arterial chemoembolization.

^a^ Alcohol group includes both MetALD and alcoholic liver disease (ALD) patients.

^b^ Defined as LR ≥ 4 (only cases with APHE) on the CT/MRI LI‐RADS v2018.

^c^ Three cases were excluded due to a lack of treatment details.

^d^ Comparison between locoregional therapy and others.

^e^ Including Sorafenib (*n* = 2), Lenvatinib (*n* = 15), and Atezolizumab + Bevacizumab (*n* = 4). All comparisons are based on a three‐group comparison among viral, c‐HCC, and Known‐HCC.

**FIGURE 4:**
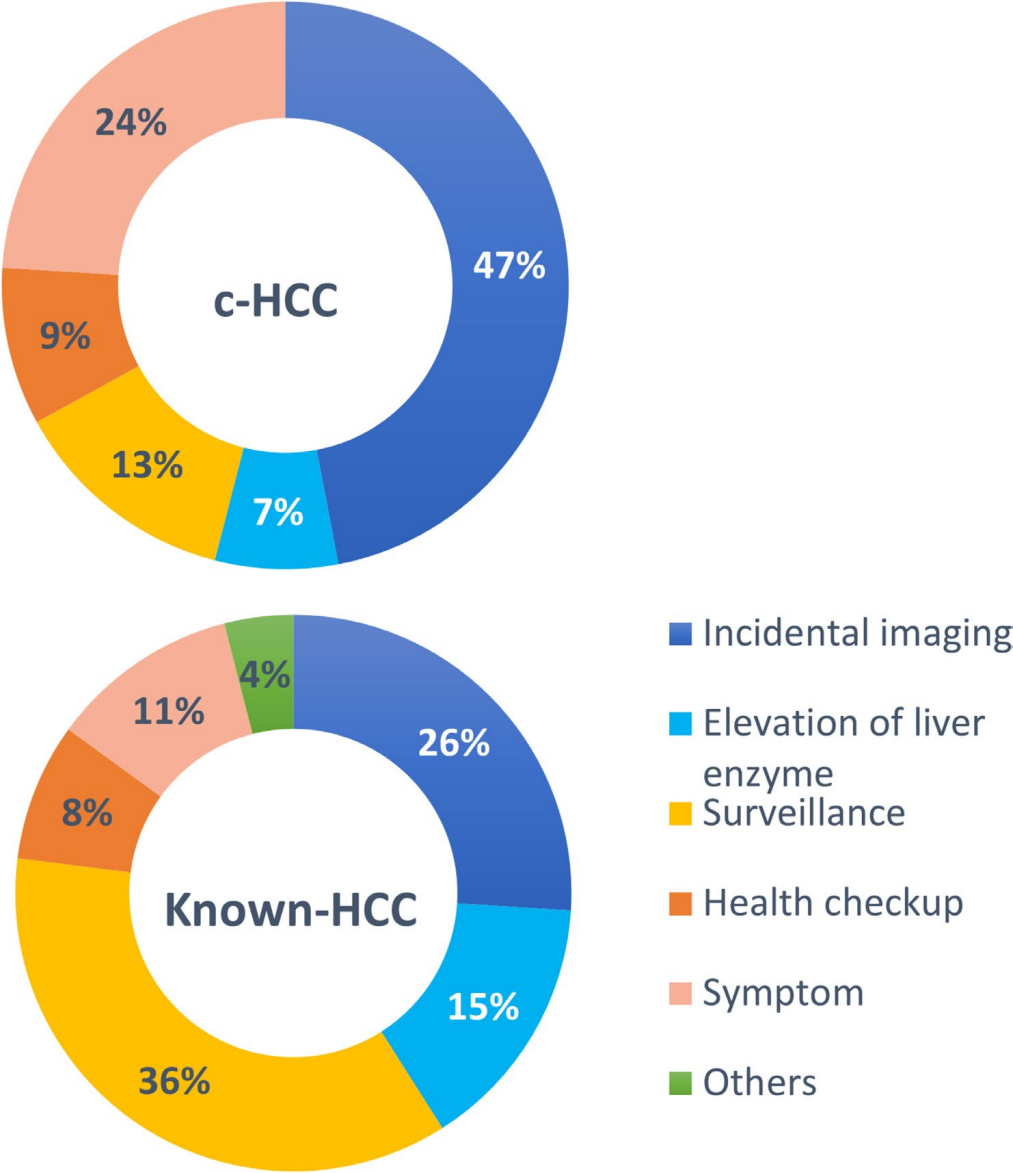
Opportunities for detection: A comparative analysis between HCC with known etiologies (Known‐HCC) and cryptogenic HCC (c‐HCC). In the cases of Known‐HCC, surveillance played a pivotal role, whereas incidental detection was a primary opportunity in c‐HCC.

**FIGURE 5:**
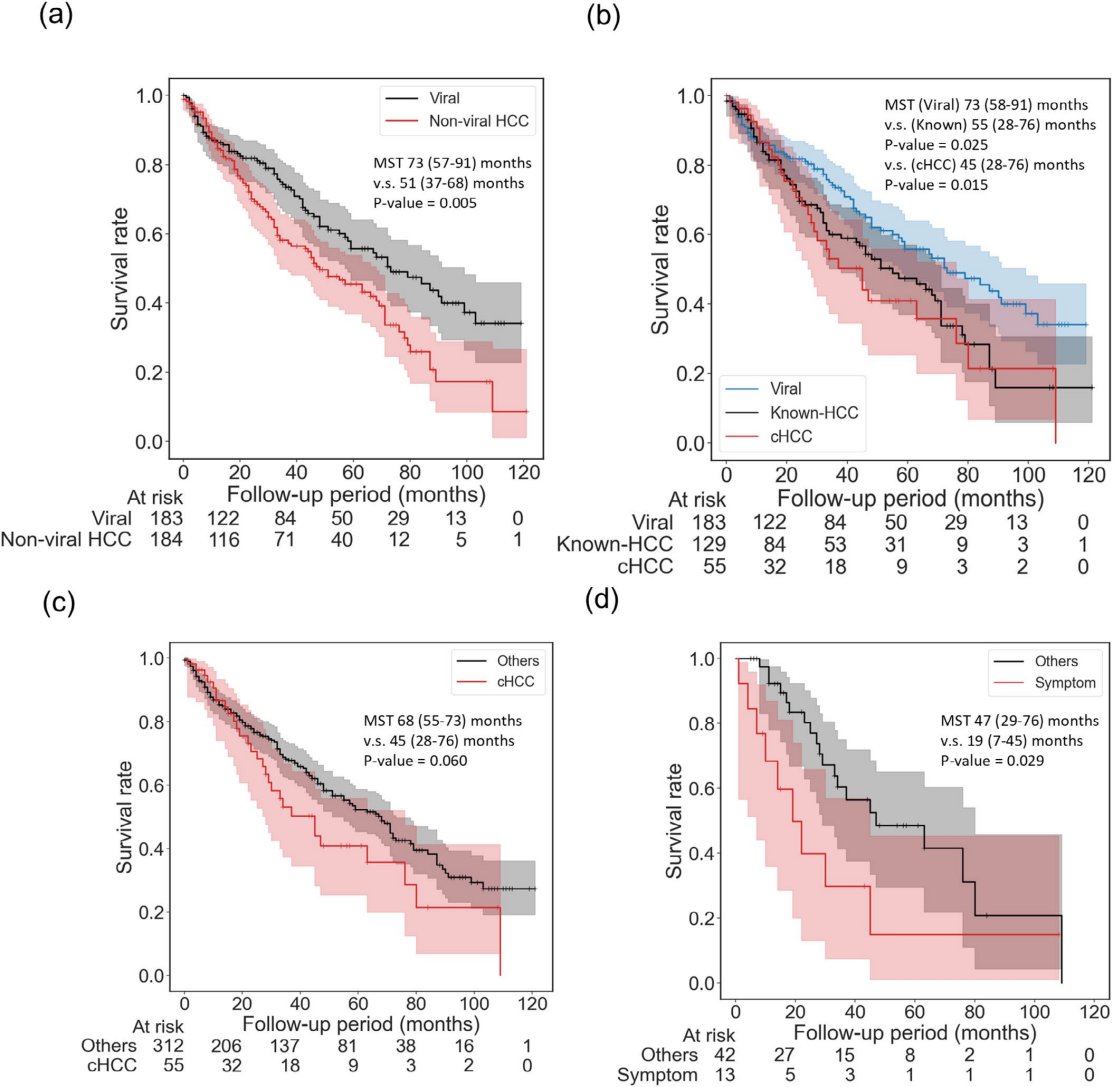
Survival analysis: (a) Comparative survival analysis between viral‐induced HCC and non‐viral HCC. The survival of non‐viral HCC was significantly poorer than the latter group (*p* = 0.005). (b) Comparative survival analysis among three groups. Patients with viral‐induced HCC demonstrated a markedly better prognosis than Known‐HCC (*p* = 0.025) and c‐HCC (*p* = 0.015). (c) Comparative survival analysis between c‐HCC and other etiologies. The survival of c‐HCC was relatively inferior to the latter group (*p* = 0.060). (d) Survival analysis comparing symptomatic cases with others regarding detection opportunities for c‐HCC. Symptomatic cases exhibited a significantly poor prognosis compared to the remaining cases (*p* = 0.029). HCC, hepatocellular carcinoma; c‐HCC, cryptogenic hepatocellular carcinoma; Known‐HCC, hepatocellular carcinoma of known etiologies.

**FIGURE 6:**
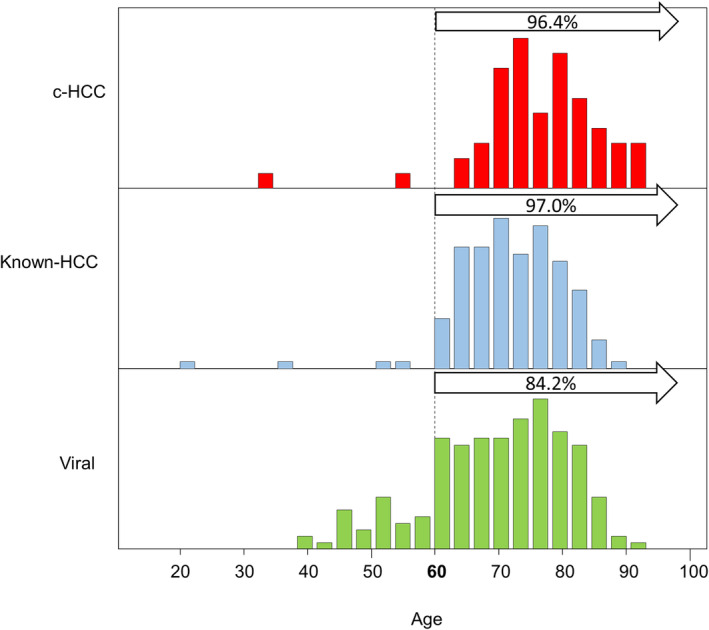
The distribution of ages: In c‐HCC and Known‐HCC, 96.4% and 97.0% were aged 60 and above, respectively. In viral‐induced HCC, 15.8% were below the age of 60.

**FIGURE 7:**
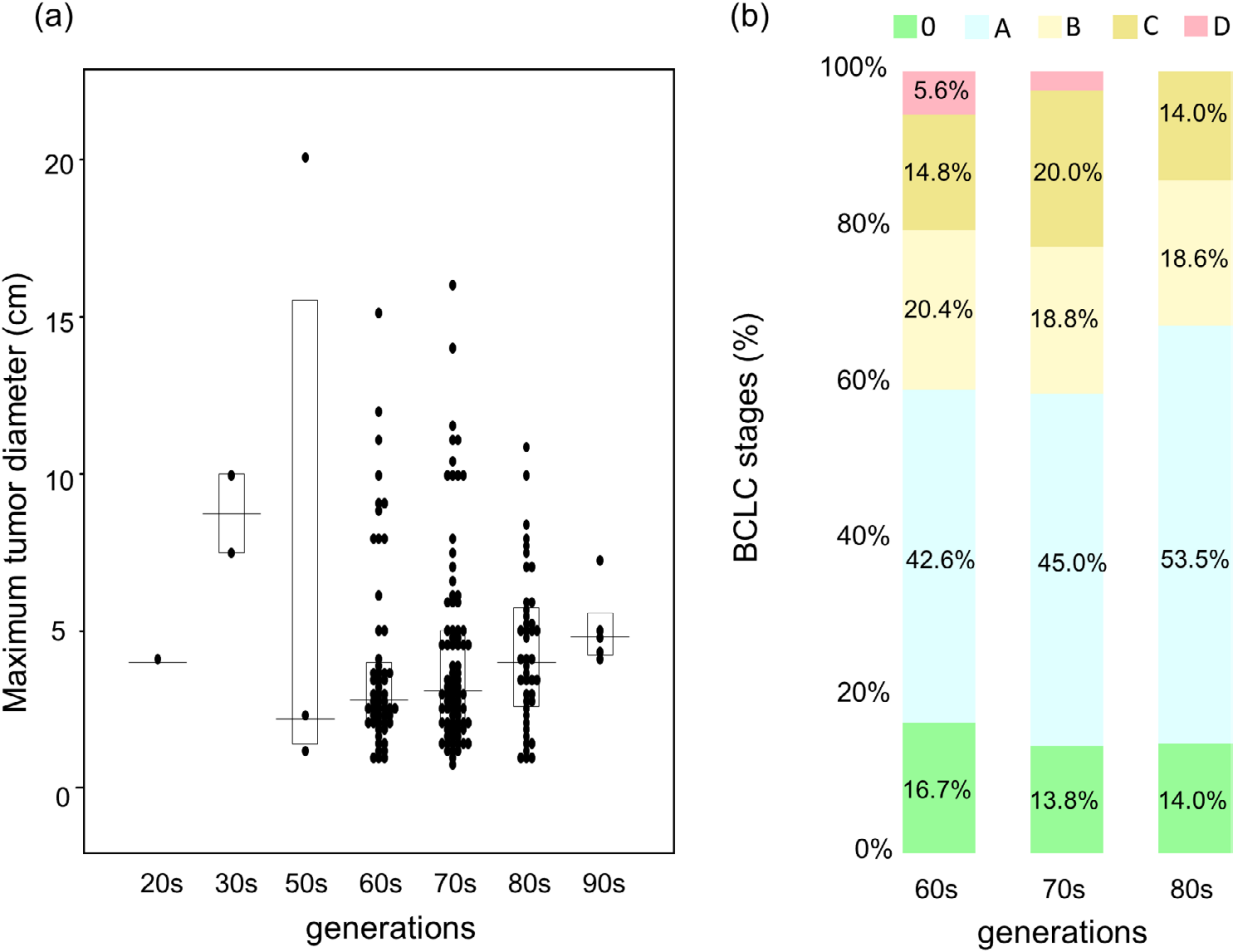
(a) The maximum tumor diameter of each generation in NBNC‐HCC: There were no statistical differences between each generation. (b) Comparison of BCLC stages in NBNC‐HCC among the 60s–80s.

**FIGURE 8:**
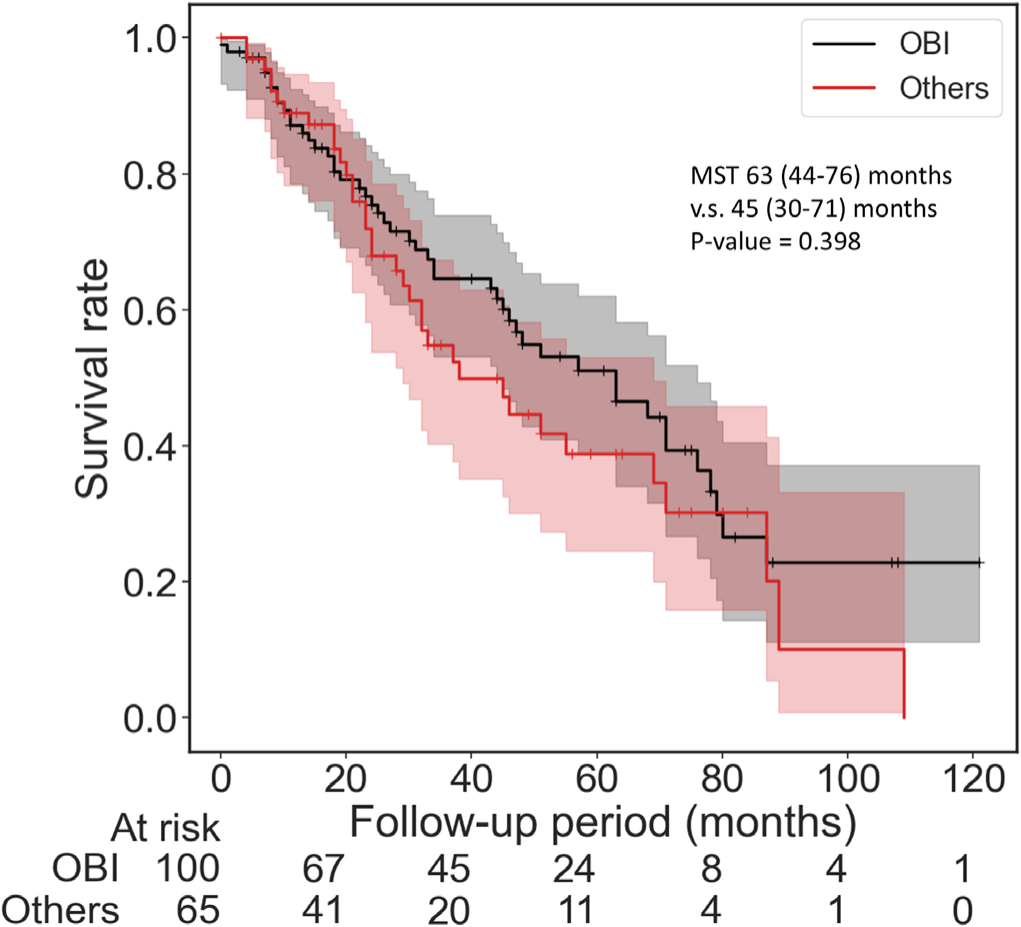
Survival analysis. Comparison between seropositive occult HBV infection (OBI) and others. There were no significant differences between the OBI and others (*p* = 0.398).

**FIGURE 9:**
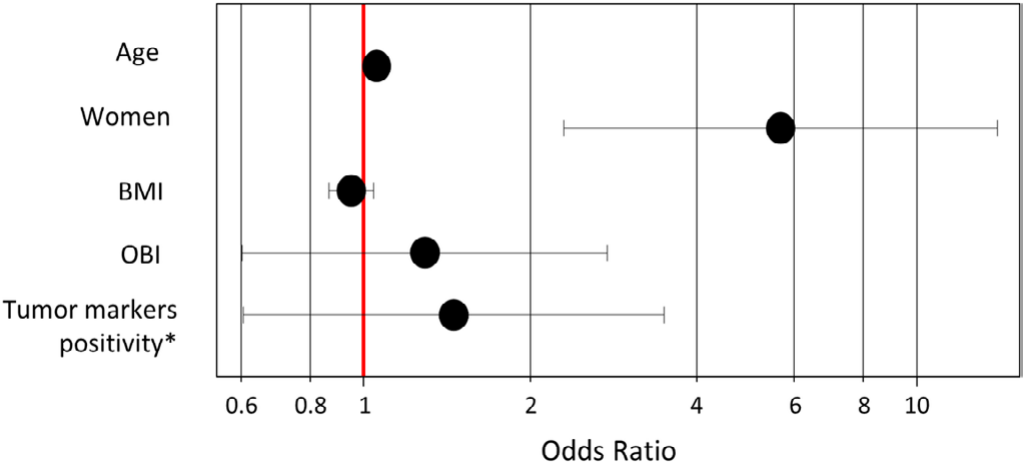
Multivariate analysis for factors associated with the c‐HCC. Age and women were associated with increased odds of c‐HCC. *Tumor markers positivity is the positivity of either alpha‐fetoprotein (AFP) or des‐gamma‐carboxy prothrombin (DCP). BMI, body mass index; OBI, occult HBV infection.

### 3.3 Opportunities for the Detection of c‐HCC

We also analyzed the opportunities for detecting c‐HCC patients compared with Known‐HCC patients (Figure 4). The opportunities for diagnosis c‐HCC were (i) surveillance (*n* = 7), (ii) elevation of liver enzyme (*n* = 4), (iii) health checkup (*n* = 5), (iv) incidentally diagnosed by imaging taken for other diseases (*n* = 26), and (v) symptom (*n* = 13) [abdominal pain (*n* = 5), back pain (*n* = 1), appetite loss (*n* = 3), epigastric discomfort (*n* = 1), abdominal mass palpation (*n* = 1), ascites (*n* = 1), and constipation (*n* = 1)]. The incidental and symptom‐based detection were twice as high in c‐HCC than in Known‐HCC, and surveillance was significantly higher in Known‐HCC (*p* = 0.001). Surveillance in c‐HCC was done for patients with cryptogenic cirrhosis. The triggers for imaging encompassed malignancy screening for DM (*n* = 4), follow‐up for previously treated cancers (lung cancer, breast cancer, thymic carcinoma, oral cancer, and hypopharyngeal cancer) (*n* = 7), precision testing for tumors (gastric subepithelial lesion, insulinoma, and renal cell carcinoma) (*n* = 3), evaluation of respiratory disease or pleural effusion (*n* = 2), follow‐up for chronic pancreatitis (*n* = 1), esophageal varices rupture (*n* = 1), renal conditions (renal failure and proteinuria) (*n* = 2), and cardiac issues (myocardial infarction, pre‐pacemaker implantation, pre‐transcatheter aortic valve implantation) (*n* = 3), and sepsis (*n* = 1). CT imaging was performed in 18 cases, while US examinations were conducted in 8 cases.

### 3.4 Survival Analysis of Patients With c‐HCC

We analyzed the survival of c‐HCC. Survival analysis was conducted on a total of 367 patients. Five cases (one in viral‐induced HCC) were excluded from the survival analysis due to the short follow‐up duration. Comparing non‐viral HCC and viral‐induced HCC, the survival of non‐viral HCC was significantly poorer than the others [73 (57.0–91.0) months vs. 51.0 (37.0–68.0) months, *p* = 0.005 / PEA: 73 (57.0–91.0) months vs. 48.0 (34.0–68.0) months, *p* = 0.003] (Figure 5a). The MST of patients with viral‐induced HCC was significantly better than Known‐HCC and c‐HCC patients [vs. 55.0 (38.0–71.0) months, *p* = 0.025 / PEA: vs. 55.0 (38.0–71.0) months, *p* = 0.015] [vs. 45 (28.0–76.0) months, *p* = 0.016 / PEA: vs. 45.0 (28.0–76.0) months, *p* = 0.016], respectively (Figure 5b). Among deceased cases, the percentage of cases attributed to other diseases did not differ significantly among the three groups (Viral, Known, and c‐HCC = 36%, 32.4%, and 31%, respectively, *p* = 0.942). There were no statistical differences between c‐HCC and Known‐HCC (*p* = 0.529). The prognosis of c‐HCC was relatively poorer than the others [45 (28.0–76.0) months vs. 68.0 (55.0–73.0) months, *p* = 0.060/PEA: vs. 45.0 (28.0–76.0) months vs. 68.0 (51.0–73.0) months, *p* = 0.072] (Figure 5c). Within the cohort of c‐HCC, the prognosis for symptomatic cases is notably unfavorable compared to other cases [Symptomatic 19.0 (7.0–45.0) months vs. Others 47.0 (29.0–76.0) months, *p* = 0.029]. (Figure 5d).

### 3.5 Distribution of c‐HCC

We further analyzed the distribution of c‐HCC compared with the other two groups (Figure 6). Most of the patients were aged 60 and older in c‐HCC (96.4%) and Known‐HCC (97.0%). This aligns with the typical age distribution observed in cancer cases, underscoring the utility of age stratification. However, two male patients under 60 years of age were outliers detected by abdominal symptoms. One patient was in his 30s with no cirrhosis and complained of abdominal pain. The maximum tumor diameter was 7.5 cm, with multiple lung and bone metastasis. He was classified into BCLC‐C. His BMI was 19.8 kg/m^2^, and he did not have any metabolic disorders, including DM, and did not demonstrate any specific etiology for carcinogenesis. Additionally, there was no family history of cancer. His transaminases were within the normal level (AST 23 U/L, ALT 29 U/L), AFP was 60,500 ng/mL, and DCP was 8086 mAU/mL on admission. The histopathological examination of the primary tumor indicated moderately differentiated HCC, whereas the bone metastasis demonstrated poorly differentiated HCC. He was treated with standard systemic chemotherapy; however, he died 4 months after the admission. The assessment of the cancer genome profile revealed the stability of microsatellite status, a tumor mutation burden of 4 mutations per megabase (Muts/Mb), alterations in TSC1 and APC, and amplification of ERBB4. However, no treatment options were deemed advisable based on the findings. Another patient was in his 50s with no cirrhosis and complained of abdominal discomfort. The maximum tumor diameter of the solitary HCC was 20 cm. He was classified into BCLC‐D. His BMI was 37.8 kg/m^2^ and had DM. He was seropositive (HBsAg−, HBcAb+) OBI. His transaminases were high (AST 187 U/L, ALT 95 U/L), total bilirubin was 12.4 mg/dL, AFP was 746 ng/mL, and DCP was 810,889 mAU/mL on admission. He could not be treated, and he died 1 month after the admission. Although his grandfather succumbed to gastric cancer, there was no suspicion of a genetic predisposition to cancer.

The first case of the two outliers in c‐HCC was difficult for early detection with no clues for enclosure. On the other hand, the second case could potentially have been identified based on metabolic risk factors, specifically obesity and DM.

Indeed, the optimal timing for tumor detection for c‐HCC remains uncertain. To address the inquiry regarding whether tumor size escalates with age and whether there exists a point of diminishing returns, we analyzed the association between tumor size and generations in non‐viral HCC (Figure 7a). While it seems that there is a gradual rise in maximum tumor size with increasing age, there was no statistically significant difference in tumor size observed across various age groups. Furthermore, we compared the distribution of BCLC stages in non‐viral HCC in the 60s to 80s. There were no differences in BCLC stages between the 60s and 80s (Figure 7b). Most cases were detected in the early to intermediate stages. It does not seem that advancing in age inherently implies an increased likelihood of reaching a point where intervention becomes too late.

### 3.6 Occult HBV Infection

Subsequently, the impact of OBI in cases of non‐viral HCC was assessed. The results of HBc‐Ab testing on 168 patients revealed seropositive OBI in 100 individuals (59.5%). The patient with congestive hepatopathy was excluded due to the absence of an HBc‐Ab test. Comparative analysis of characteristics between the seropositive OBI group and the remaining patients indicated no significant differences (Table 2). Survival analysis was conducted on a total of 165 patients. Three cases (one in OBI) were excluded from the survival analysis due to the short follow‐up duration. Additionally, survival outcomes showed no noteworthy distinction between the seropositive OBI group and the remaining patients [63 (44.0–76.0) months vs. 45 (30.0–71.0) months, *p* = 0.398 / PEA: 63.0 (44.0–76.0) months vs. 45.0 (30.0–71.0) months, *p* = 0.454] (Figure 8). These findings collectively suggest that OBI does not exert a remarkable influence on non‐viral HCC.

**TABLE 2:** Occult HBV infection in non‐viral HCC.

Characteristics	Non‐viral HCC (*n* = 168)		*p*
	Seropositive OBI (*n* = 100)	Others (*n* = 68)	
Men/Women	79: 21	56: 12	0.591
Age (years)	73 (62.0–89.0)	73.0 (40.0–90.8)	0.474
BMI	23.9 (17.4–37.8)	24.3 (18.0–31.9)	0.733
Alcohol^a^	51	41	
MASLD/MASH	9	8	
AIH	2	1	0.548
PBC	1	0	
PSC	0	0	
Cryptogenic	37	18	
Liver status			
Cirrhosis	34 (34.0%)	31 (45.6%)	0.130
CP‐A	20	19	
CP‐B	13	10	0.736
CP‐C	1	2	
Tumor markers			
AFP	8.3 (2.0–15672.0)	6.4 (1.4–71063.2)	0.518
DCP	166.0 (10.0–227710.0)	84.5 (10.2–19244.8)	0.124
Tumor size (cm)	3.5 (1.1–14.0)	3.0 (1.0–10.9)	0.310
Imaging diagnosis			
Typical HCC^**b**^	97 (97.0%)	67 (98.5%)	0.523
Histologically proven HCC	59	35	
Well‐differentiated	20	9	
Moderately differentiated	36	25	0.577
Poorly differentiated	3	1	
BCLC staging			
0	11	13	
A	48	33	
B	19	11	0.582
C	19	9	
D	3	2	
TNM staging			
IA	12	13	
IB	33	26	
II	32	15	
IIIA	15	8	0.611
IIIB	2	2	
IVA	0	0	
IVB	5	4	

*Note:* Data expressed as median (95% CI) or mean ± SD.

Abbreviations: AFP, alpha‐fetoprotein; ALBI, albumin‐bilirubin; BCLC, Barcelona Clinic Liver Cancer; BMI, body mass index; BSC, best supportive care; CP, Child‐Pugh classification; CR, complete response; DCP, des‐gamma‐carboxyprothrombin; DM, diabetes mellitus; HAIC, hepatic artery infusion chemotherapy; HBV, hepatitis B virus; HCC, hepatocellular carcinoma; HCV, hepatitis C virus; MASLD/MASH, metabolic dysfunction‐associated steatotic liver disease/steatohepatitis; RFA, radiofrequency ablation; SBRT, stereotactic body radiotherapy; TACE, trans‐arterial chemoembolization.

^a^ Alcohol group includes both MetALD and alcoholic liver disease (ALD) patients.

^**b**^ Defined as LR ≥ 4 (only cases with APHE) on the CT/MRI LI‐RADS v2018.

### 3.7 Multiple Regression Analysis

Moreover, a multiple regression analysis was performed in the entire non‐viral patient cohort, excluding those lacking OBI data (*n* = 168). The analysis included age, sex, OBI, and the positivity of either AFP or DCP as variables to identify independent factors linked to c‐HCC. The findings reveal that both age and women are associated with increased odds of c‐HCC (Table 3) (Figure 9).

**TABLE 3:** Multivariate regression analysis of factors associated with c‐HCC.

Factors	Multivariate analysis					
	*β*	SE (*β*)	*z*	OR	95% CI	*p*
Age	0.05214	0.02403	2.170	1.054	1.054–1.104	0.030
Women	1.734	0.4609	3.762	5.664	5.664–13.978	< 0.001
BMI	−0.05343	0.04693	−1.139	0.948	0.948–1.039	0.255
OBI	0.2529	0.3878	0.652	1.288	1.288–2.754	0.514
Tumor markers positivity^a^	0.3731	0.4466	0.835	1.452	1.452–3.485	0.404

Abbreviations: BMI, body mass index; CI, confidence interval; c‐HCC, cryptogenic HCC; OBI, occult HBV infection; OR, odds ratio; SE, standard error.

^a^ Tumor markers positivity is the positivity of either alpha‐fetoprotein (AFP) or des‐gamma‐carboxy prothrombin (DCP).

## 4 Discussion

This study unveiled that the characteristics of c‐HCC, exhibiting a gradual increase in recent years, are predominantly observed in elderly individuals without cirrhosis and DM. Detection often relied on incidental imaging.

Approximately 70% of these cases were incidentally depicted by imaging for other concomitant diseases or identified by symptom. Notably, c‐HCC exhibited a significantly larger maximum tumor size compared to other HCC subtypes, and either AFP or DCP markers were found to be positive in these cases. Most c‐HCC cases do not exhibit cirrhosis, and a notable proportion do not present with diabetes. Furthermore, the survival outcomes for patients with c‐HCC were significantly poorer than those with viral‐induced HCC but comparable to Known‐HCC. Nevertheless, despite the advanced stage of c‐HCC, the preserved hepatic function suggested that chemotherapy could potentially enhance the prognosis in advanced cancer cases.

The variations observed in tumor size, staging, and tumor marker positivity between c‐HCC and Known‐HCC indicate differences in the opportunity for surveillance. Patient demographics showed similarities, except for the lower prevalence of cirrhosis in c‐HCC cases. Furthermore, the markedly higher platelet counts in c‐HCC may be linked to a diminished incidence of cirrhosis. As a result, relying on platelet count and liver stiffness assessment proves to be less efficacious in detecting c‐HCC.

Our analysis did not reveal statistically significant differences in maximum tumor diameter within each generation group. In essence, advancing age does not inherently correlate with larger tumor size. Nonetheless, it remains a contention that tumor size may vary depending on whether the patient is under active surveillance.

This study classified seropositive OBI (HBc‐Ab+) as OBI, with no HBV‐DNA measurements conducted in patients with HBc‐Ab negativity. Consequently, seronegative OBI cases were not excluded from the analysis. However, seronegative OBI, if present, would likely be exceedingly rare. The positive ratio of serological OBI in c‐HCC was relatively high (59.5%) compared to other reports from Japan (around 30%) [26]. Some reports suggest an association between OBI and HCC carcinogenesis [26, 27]. OBI is suspected as a sub‐entity within the spectrum of c‐HCC. Nevertheless, its precise role in carcinogenesis remains less well‐defined [8, 10, 28, 29]. Omichi et al. defined the “pure NBNC group” in resected cases as patients negative for all three serologic markers: HCV‐Ab, HBs‐Ag, and HBc‐Ab [30]. In this study, the definition criteria for c‐HCC are considerably more stringent than theirs, as it excludes all known etiologies. They indicated that patients with a history of HBV infection (HBc‐Ab +) among non‐viral HCC patients might have better survival outcomes after complete resection of HCC than patients with others. This is not consistent with our results. However, in our study, even among cases of seropositive OBI, no apparent substantial impact on the prognosis of non‐viral HCC was evident. The present study included all cases, while their report only included resected cases. Moreover, in the present study, OBI was not identified as an independent factor for c‐HCC in the multivariate analysis of the non‐viral cohort. While the frequency of OBI may be higher than that in the general population, the present results do not allow us to conclude whether OBI is implicated in the carcinogenesis of c‐HCC. Consequently, the utility of OBI screening in detecting c‐HCC remains uncertain.

Moreover, it remains possible that congenital or genetic diseases affecting the liver have not been completely ruled out in the c‐HCC cases, highlighting a limitation in the term “cryptogenic.” However, the c‐HCC cohort did not include patients with obvious liver dysfunction, at least from childhood, nor did it include cases of suspected Wilson's disease. For instance, conditions such as alpha‐1 antitrypsin deficiency, which can be problematic in Western populations, are rare in Japan, with an estimated prevalence of 2.03 to 2.08 cases per 10 million people. Therefore, we consider the possibility of these diseases being involved in this cohort to be quite limited.

Ho et al. conducted a recent analysis on a substantial cohort of 2937 patients in Taiwan, revealing an incidence rate of 20.4% for c‐HCC, excluding known etiologies [31]. In our cohort, the incidence was 14.8%, demonstrating a comparable rate. Their observations highlighted that c‐HCC cases were often older, with lower serum ALT levels and higher platelet counts than viral‐induced HCC traits consistent with our c‐HCC cases. Their cases also shared similarities with ours in terms of larger tumor sizes, advanced cancer stages, and the administration of non‐curative treatments. However, the median overall survival of their c‐HCC cases (19 months) was notably shorter than that of our cases (45 months). The difference might be due to their cohort's high rate of BSC cases (19%). The survival rate is the same as that of symptomatic cases in our cohort. The opportunities for detection were not indicated in the manuscript. While they discuss prognostic factors, it is evident that the poor prognosis results from late detection. Regarding c‐HCC, we contend that it is more crucial to explore how to detect it rather than delve into prognostic factors.

The findings of this study highlight that patients detected after the onset of symptoms face a notably grim prognosis. In contrast, those who can undergo specific diagnostic tests (such as blood tests and coincidental imaging examinations) experience a relatively favorable prognosis. Despite the inherent difficulties in discerning c‐HCC, primarily attributable to the absence of unequivocal diagnostic indicators, it is imperative to emphasize that c‐HCC exhibits a near‐exclusive prevalence among individuals aged 60 years and above. In this viewpoint, c‐HCC might be able to consider an aging‐related HCC.

Additionally, women's dominancy was extracted as an independent factor for c‐HCC. Globally, the reported incidence of HCC is two to three times higher in men, attributed to established risk factors like alcohol consumption, sex steroid hormones, immune response, and potential epigenetic differences between men and women. In 2017, Valery et al. anticipated a decrease in Japanese HCC cases for both genders, particularly noting a decline in women [32]. However, a recent comprehensive report on HCC in Japan (*n* = 20,547, 1996–2019) revealed no distinct trend by gender over time [4]. In our current study, the gender ratio for c‐HCC is 34 vs. 21, indicating a 1.6‐fold higher prevalence in men. However, this difference increases to 2.4 times for the virus and 8.5 times for Known‐HCC. Despite these variations, the gender difference in c‐HCC is relatively small. This might have influenced the identification of women as an independent factor. Eliminating various known risk factors, including alcohol consumption, provides a more accurate perception that the difference between men and women in HCC carcinogenesis has narrowed. Therefore, we conclude that c‐HCC should be considered as posing approximately the same risk for both men and women.

The statistical data from 2019 revealed that 37,296 individuals in Japan received a diagnosis of liver cancer annually [33]. Within this cohort, there were 34,295 patients aged 60 and above. According to our study, 15% of these cases are categorized as c‐HCC, and the projected annual count of c‐HCC cases would be 5144. About 70% of c‐HCC cases are incidentally discovered through imaging studies or clinical presentations. The unexcavated c‐HCC is estimated to be 3600 cases annually. The demographic of individuals in Japan aged over 60 is approximately 43.74 million, with approximately 60% of them undergoing health checkups [34]. Consequently, the remaining 40% are deemed eligible for c‐HCC surveillance. Accordingly, 12.25 million out of the 17.5 million annual patients aged 60 or older will be the focus of c‐HCC surveillance. Therefore, the anticipated annual incidence of c‐HCC in this subgroup is estimated to be 0.029%.

Parikh et al. also reported that an HCC incidence > 0.4% per year and surveillance adherence > 19.5% biannually was necessary for US and AFP to be cost‐effective compared to no surveillance [35]. The estimated incidence of 0.029% is deemed insufficient for surveillance, being approximately tenfold lower. Consequently, a biannual (0.5‐year) frequency is considered excessive. The interval is tentatively estimated at 5 years, based on a straightforward calculation.

The AASLD Practice guidance updated 2023 recommends surveillance in cirrhotic patients of any etiology (Child‐Pugh A–B) at high risk of developing HCC (incidence of HCC ≥ 1.0% per year), cirrhotic patients (Child‐Pugh stage C), and non‐cirrhotic chronic hepatitis B (incidence of HCC ≥ 0.2% per year). It recommends against it in patients with life‐limiting comorbid conditions, with HCV infection post SVR with advanced fibrosis but without cirrhosis, and with NAFLD with advanced fibrosis without cirrhosis [36].

The EASL Clinical Practice Guidelines published in 2018 recommends surveillance in cirrhotic patients (Child‐Pugh stages A and B), cirrhotic patients (Child‐Pugh stage C) awaiting liver transplantation, non‐cirrhotic HBV patients at intermediate or high risk of HCC (according to PAGE‐B classes for Caucasian subjects, respectively10–17and ≥ 18 score points), and non‐cirrhotic F3 patients, regardless of etiology may be considered for surveillance based on an individual risk assessment [37]. For chronic hepatitis B (CHB), PAGE‐B is a practical risk score that includes platelet count, age, and sex and has been validated in various patient populations [38, 39, 40, 41]. PAGE‐B is certainly for predicting HCC in CHB patients; however, interestingly, 85.5% of c‐HCC scores at least 10 points only by age (60–69 = 8, ≥ 70 = 10). This demonstrates the significant influence of aging on the process of carcinogenesis.

The conventional HCC surveillance system is unsuitable for detecting c‐HCC. Moreover, the concept of “enclosure” or “surveillance” may not be applicable to c‐HCC. Given the cost implications, implementing public policy‐driven screenings is economically unviable.

The “Nara Declaration 2023: Stop Chronic Liver Disease (CLD)” was unveiled at the 2023 Annual Meeting of the Japan Society of Hepatology in Nara, calling for medical consultation for individuals with ALT levels exceeding 30 IU/L. However, among cases of c‐HCC, 60% exhibited ALT levels below 30 IU/L. Ho et al. also indicated lower levels of ALT in c‐HCC [31]. While emphasizing the significance of identifying elevated ALT values, it implies that over half of the cases of c‐HCC may go undetected. Hence, a practical recommendation involves advising elderlies to undergo comprehensive health checkups, regardless of whether their ALT is abnormal or exceeds 30 IU/L.

Consequently, our current proposal for the detection of c‐HCC is as follows: (1) Elderly individuals aged 60 years or older are advised to undergo comprehensive health checkups, including abdominal imaging, at least once every 5 years, irrespective of specific risk factors. (2) Additional assessment, including abdominal imaging examinations, is recommended in cases of elevated liver enzymes. (3) Individuals with cryptogenic cirrhosis (Child‐Pugh A‐B) should undergo semiannual surveillance every 6 months.

The present study has limitations. This was a single‐center retrospective study with a limited sample size. However, our community (Tottori prefecture) is a characteristic area in the super‐high‐aged society. The percentage of the population aged 65 and over in our community was 32.0% in 2022. In 2020, the percentage of the population aged 65 and over in Japan was 28.6%, exceeding the U.S.A. (16.6%), Sweden (20.3%), France (20.8%), Germany (21.7%), and Italy (23.3%) [42]. WHO estimates that, by 2050, two‐thirds of the world's population over 60 years will live in low‐ and middle‐income countries [43]. Therefore, even with this small sample size, it is possible to reflect the future landscape surrounding HCC. We are also currently in the process of establishing partnerships with other institutions and preparing the necessary protocols for multicenter data collection. Approximately half of the HCC cases were not confirmed by histology. While histological evaluation is crucial for an accurate diagnosis, performing a biopsy can be challenging in some cases due to factors such as ascites or coagulopathy. To address this, we relied on the LIRADS criteria, including only typical HCC cases with an LR of ≥ 4, which is practical for real‐world clinical settings. Furthermore, this study involves unavoidable selection bias. Certainly, accurate delineation of the characteristics of the population at risk of c‐HCC cases requires a strict comparison with non‐HCC patients through comparative analysis; however, this challenge possesses the inherent difficulty of accurately defining “non‐HCC” patients as a control group. The term means a patient who “never” developed HCC at any time in his life. Currently, no comprehensive database covering all HCC risk factors, validated by imaging studies to confirm the absence of HCC development during a patient's lifetime, exists. Therefore, establishing a comparison target group is not feasible. As with other cancers, when the cause is unknown, the focus shifts to investigating the characteristics of as many c‐HCC cases as possible. In this sense, I believe this research holds significance.

## 5 Conclusions

The characteristics of c‐HCC patients included elderly individuals without cirrhosis or diabetes, with minimal gender differences. Detection was frequently incidental through imaging. Considering the limitations of conventional surveillance, it seems reasonable to propose that abdominal imaging be included in cancer screening, particularly for individuals aged 60 and older. The continual rise in the aging population implies that the c‐HCC will play a more significant role in the future.

## Author Contributions

**Takaaki Sugihara:** conceptualization (lead), data curation (lead), formal analysis (lead), investigation (lead), methodology (lead), project administration (lead), resources (lead), software (lead), visualization (lead), writing – original draft (lead). **Takakazu Nagahara:** conceptualization (equal), investigation (equal), project administration (equal), writing – review and editing (equal). **Takuya Kihara:** data curation (supporting), investigation (supporting). **Suguru Ikeda:** data curation (supporting), writing – review and editing (supporting). **Yoshiki Hoshino:** writing – review and editing (supporting). **Yukako Matsuki:** data curation (supporting), investigation (supporting), writing – review and editing (supporting). **Takuki Sakaguchi:** writing – review and editing (supporting). **Hiroki Kurumi:** writing – review and editing (supporting). **Takumi Onoyama:** data curation (supporting), formal analysis (supporting), software (supporting), visualization (supporting), writing – review and editing (supporting). **Tomoaki Takata:** writing – review and editing (supporting). **Tomomitsu Matono:** investigation (supporting), resources (supporting), writing – review and editing (supporting). **Hajime Isomoto:** supervision (supporting), writing – review and editing (supporting).

## Ethics Statement

The Tottori University Hospital Institutional Review Board approved the study protocol under the 1975 Declaration of Helsinki guidelines. We applied the Opt‐out method to obtain consent for this study.

## Conflicts of Interest

The authors declare no conflicts of interest.

## Data Availability

The data that support the findings of this study are available on request from the corresponding author. The data are not publicly available due to privacy or ethical restrictions.
